# CAVD, towards better characterization of void space for ionic transport analysis

**DOI:** 10.1038/s41597-020-0491-x

**Published:** 2020-05-22

**Authors:** Bing He, Anjiang Ye, Shuting Chi, Penghui Mi, Yunbing Ran, Liwen Zhang, Xinxin Zou, Bowei Pu, Qian Zhao, Zheyi Zou, Da Wang, Wenqing Zhang, Jingtai Zhao, Maxim Avdeev, Siqi Shi

**Affiliations:** 1 School of Computer Engineering and Science, Shanghai University, Shanghai, 200444 China; 2 State Key Laboratory of Advanced Special Steel, School of Materials Science and Engineering, Shanghai University, Shanghai, 200444 China; 3 Materials Genome Institute, Shanghai University, Shanghai, 200444 China; 4 Department of Physics and Shenzhen Institute for Quantum Science & Technology, Southern University of Science and Technology, Shenzhen, 518055 China; 5 School of Materials Science and Engineering, Guilin University of Electronic Technology, Guilin, 541004 China; 6 Australian Nuclear Science and Technology Organisation, Locked Bag 2001, Kirrawee DC, NSW 2232 Australia; 7 School of Chemistry, The University of Sydney, Sydney, 2006 Australia

**Keywords:** Batteries, Computational methods

## Abstract

Geometric crystal structure analysis using three-dimensional Voronoi tessellation provides intuitive insights into the ionic transport behavior of metal-ion electrode materials or solid electrolytes by mapping the void space in a framework onto a network. The existing tools typically consider only the local voids by mapping them with Voronoi polyhedra vertices and then define the mobile ions pathways using the Voronoi edges connecting these vertices. We show that in some structures mobile ions are located on Voronoi polyhedra faces and thus cannot be located by a standard approach. To address this deficiency, we extend the method to include Voronoi faces in the constructed network. This method has been implemented in the CAVD python package. Its effectiveness is demonstrated by 99% recovery rate for the lattice sites of mobile ions in 6,955 Li-, Na-, Mg- and Al-containing ionic compounds extracted from the Inorganic Crystal Structure Database. In addition, various quantitative descriptors of the network can be used to identify and rank the materials and further used in materials databases for machine learning.

## Introduction

All-solid-state batteries are the promising candidates for electric vehicles^1^, smart grid^2^ and other electrochemical power sources, due to their high safety, long cycle life and high energy density^3–6^. One of the prerequisites for both inorganic solid electrolytes and electrodes is high ionic conductivity, which requires a connected mobile ions pathways. Currently, widely used computational approaches to predict or simulate ionic conduction pathways include both *ab initio* and empirical methods. The methods based on the first-principles calculations include *ab initio* molecular dynamics^7–9^ and the first-principles nudged elastic band method (FP-NEB)^10^. They provide high accuracy but they are limited by high computational cost and often involve complicated manual pre- and post-processing of the input data and results. At the opposite side of the spectrum, the low-cost empirical methods, such as geometric analysis^11–15^ and bond valence method (BV)^16–21^, have gained popularity in high-throughput screening^22–29^ and obtaining preliminary insights quickly for further accurate calculations. In this context, we have developed a high-throughput screening platform^30^ that integrates material database with hierarchical ion-transport calculations realized by implementing empirical algorithms to assist in FP-NEB calculation. To meet the requirements of our platform for automated unsupervised workflow, we also independently developed the bond valence site energy (BVSE) calculation program (has been reported in our paper^31^) and the geometry-based ion-transport analysis library CAVD (presented in this paper).

In the geometric model considering atoms as spheres of characteristic radii, the crystal space can be divided into two complementary subspaces: the subspace occupied by atoms and that of interatomic voids^32^. The atom subspace can be characterized by the composition, symmetry, topology, and geometry, which enables efficient classification of structures. On the other hand, the analysis of the void subspace of crystal structures allows to identify materials favorable for transport of ions or molecules, e.g. in solid electrolytes and metal organic frameworks, respectively^33^.

Current structure analysis tools, including PLATON^34^, ToposPro^35^ and Zeo++^22^, have already shown the ability to analyze the void space^22,32,33,36^. PLATON provides a number of techniques to detect and handle voids in crystal structures by grid method. Based on the Voronoi theory, both ToposPro and Zeo++ characterize the void space as a 3D Voronoi network^37^ with Voronoi polyhedra vertices and edges indicating the centers of the local cavities and pathways between them, respectively. Although these tools have been successfully used for void space analysis, some improvements are still desirable. For example, since accuracy of the grid method depends on the grid density, the computational cost of the method very rapidly increases with the grid resolution. The Voronoi approach implemented in ToposPro and Zeo++ only identifies the largest cavities in the local environment, however the experimentally determined positions of the mobile atoms which are not close enough to those Voronoi vertices cannot be easily automatically associated with them, which in turn complicates automatic search of transport pathways. In addition, the standard Voronoi decomposition (also known as Voronoi-Dirichlet partition)^38^ adopted by ToposPro does not consider the atomic radii that affects the simulation results for polydisperse particle systems^39^. The radical Voronoi decomposition^40^ and the ability to consider radii are implemented in Zeo++, but its built-in radius table and radius assignment scheme do not take into account dependence for the ionic radii on different coordination environments.

Inspired by Zeo++ and ToposPro, we developed a high-throughput ion-transport analysis tool CAVD based on the radical Voronoi decomposition. To address the challenge in the calculation of ionic radius calculation, we calculate the ionic radius by combining the rigorous definition of coordination number proposed by O’Keeffe^41,42^ and the table of the effective ionic radii of Shannon^43^. Moreover, we consider the Voronoi polyhedra faces as the possible sites for mobile ions. In our tool, the void space is characterized as the 3D network consisting of interstices, channels between interstices, and bottlenecks of the channels, which are identified and quantified from the position and geometry of Voronoi polyhedra vertices, edges, and faces, respectively. The geometric and topological parameters of the network are then translated into ion transport descriptors. These descriptors form the foundation of machine-learning algorithms to predict and optimize materials properties.

All calculations discussed in this work were performed with the developed python package CAVD, which calls the libraries Spglib^44^ and Zeo++ (based on a modification of Voro++^45^ software library) for symmetry analysis and periodic radical Voronoi decomposition, respectively. The code can be accessed in the repository (https://gitee.com/shuhebing/cavd) based on the FAIR principles for the benefit of the broad research community.

## Methods

**Fig. 1:**
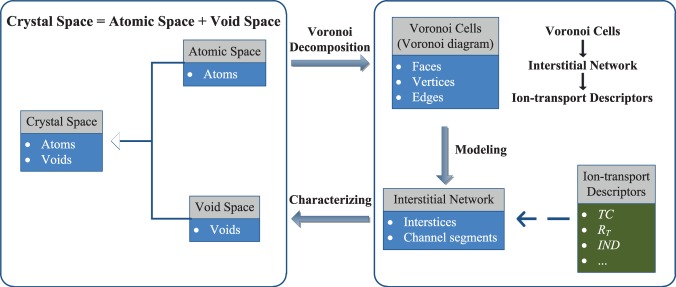
The class diagram of objects that are involved in our tool.

The Voronoi decomposition has been widely used to construct a graph representation of the void space for a given arrangement of atoms in a periodic domain^37,38,46,47^. The resulting Voronoi diagram is composed of Voronoi cells, characterized by vertices, edges and faces. The Voronoi faces are the perpendicular planes between two neighboring framework atoms, and the vertices and edges of the Voronoi cell are by definition at the largest possible distance from the framework atoms around them. The Voronoi network, built of Voronoi vertices and edges, maps the void space in the framework. Based on these features, the procedures of our method are summarized as following (Fig. 1):

1. Perform Voronoi decomposition of the void space in the framework;
2. Define an interstitial network for ion transport analysis;
3. Define and calculate ionic transport descriptors.

### Voronoi algorithms for void space characterization

The standard Voronoi decomposition proposed by Georgy Voronoi^48^ is a mathematical approach to dividing space^38,49^. In a finite-dimensional Euclidean space *Z*, if the Euclidean distance between generator (e.g., point, circle or sphere) *p* and *p*_*i*_ (*i* = 1,2,…,*n*) is denoted by *d*(*p*, *p*_*i*_), the standard Voronoi cell^49^ corresponding to *p*_*i*_ satisfies

1 $$V({p}_{i})=\bigcap _{j\ne i}\left\{p\in Z\left|d(p,{p}_{i}) < d\left(p,{p}_{j}\right)\right.\right\},j\in \{1,2,\ldots ,n\}.$$

The Voronoi diagram $V=\left\{V({p}_{1}),V({p}_{2}),\ldots ,V({p}_{n})\right\}$ formed by $P=\{{p}_{1},{p}_{2},\ldots ,{p}_{n}\}\,(n\ge 2)$ is thus a division of space *Z*. Each Voronoi cell is a convex polyhedron, which is obtained by constructing the perpendicular bisector (for monodisperse system) planes between two neighboring generators. Replacing the generators with ions, the Voronoi cell can be used to describe the local environment in an ionic crystal structure. The Voronoi vertices can be used to describe the sites of local largest voids, and the Voronoi edges refer to the least restrictive paths from one local largest void to the next because they are farthest away from the ions around themselves.

**Fig. 2:**
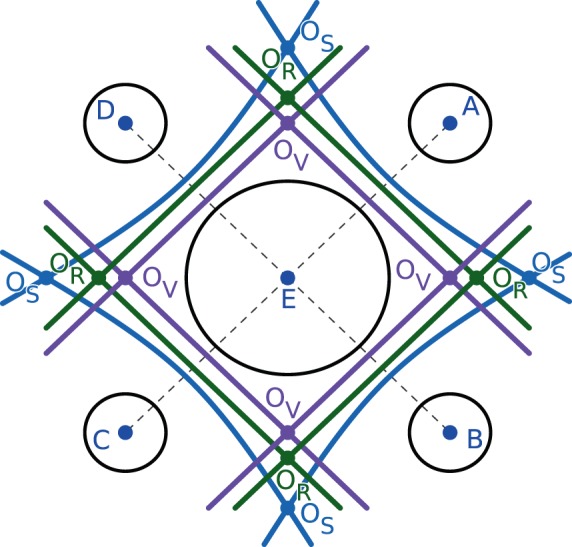
The standard Voronoi cell, Voronoi S cell and radical Voronoi cell of circle E in 2D space. The boundaries of standard Voronoi cell (purple lines) and radical Voronoi cell (green lines) are straight line, but the boundary of Voronoi S cell (blue lines) is a hyperbolic curve. In the local environment formed by circle C, D and E, the center of local largest void coincides with O_S_, and the deviation of O_R_ from O_S_ is less than the deviation of O_V_ from O_S_.

However, the standard Voronoi cell constructed by bisecting the interatomic distances is not adequate for structures with atoms of different size (see O_V_ in Fig. 2). The difference in radii can be taken into account in a so called Voronoi S decomposition^50–52^, as following:

2 $$V({p}_{i})=\bigcap _{j\ne i}\left\{p\in Z\left|d(p,{p}_{i})-{r}_{i} < d(p,{p}_{j})-{r}_{j}\right.\right\},j\in \{1,2,\ldots ,n\}.$$

However, the curved boundaries (see O_S_ in Fig. 2) of the Voronoi S cell obtained by Eq. (2) are computationally difficult to handle, and the radical Voronoi decomposition^40,53–56^ is chosen as a compromise between the accuracy and efficiency^57,58^:

3 $$V({p}_{i})=\bigcap _{j\ne i}\left\{p\in Z\left|d{(p,{p}_{i})}^{2}-{r}_{i}^{2} < d{(p,{p}_{j})}^{2}-{r}_{j}^{2}\right.\right\},j\in \{1,2,\ldots ,n\}.$$

The radical Voronoi cell constructed by radical Voronoi decomposition has the following three main features:

- (1) The radical Voronoi cell is a convex polyhedron.
- (2) The overall shape of radical Voronoi cell is similar to Voronoi S cell.
- (3) The radical Voronoi cell will degenerate into standard Voronoi cell when *r*_*i*_ = *r*_*j*_.

The radical Voronoi decomposition is easy to implement and provides more adequate description of the void space than standard Voronoi decomposition for a structure with atoms of unequal radii. Although the vertices of the radical Voronoi cell may no longer precisely coincide with the centers of local maximally large interstices (see O_R_ in Fig. 2), previous studies^40,57,58^ and our derivation (S1 of the Supplementary Information) demonstrates that it has little effect on ionic transport analysis.

### Interstitial network for ionic transport analysis

For the analysis of ionic transport, it is necessary to characterize the void space formed by the framework atoms and then determine the subspace which mobile ions can access^33,59^. The Voronoi network, which maps the void space in the packing of framework atoms by Voronoi decomposition, identifies the interstices and the channel topology. Since the Voronoi vertex may be expected to be a local low-energy position (since it maximizes the distance to its coordinating atoms)^60^, the potential mobile ion diffusion path can be further evaluated by determining the parts of this network through which the mobile ions could theoretically migrate. A set of local geometrical descriptors, *r*_SD_, *r*_Chan_^13,23^ and *G*_3_^61–63^ has been introduced for the evaluation of ionic transport in previous work. *r*_SD_ is the radius of a spherical domain whose volume equals to the secondary Voronoi cell around the vertices of the primary Voronoi cell. *r*_Chan_ describes the channel size, which is the radius of a circle passing through the bottleneck area constituted by three framework atoms. *G*_3_ is the second moment of inertia of the Voronoi cell, which is necessary to filter out unreasonable *r*_SD_ values caused by the severe distortion of the secondary Voronoi cell. However, for these local descriptors, Voronoi decomposition needs to be performed twice, and the value of *r*_Chan_ is affected by the distortion of the bottleneck region. Moreover, two different criteria are required to determine the accessibility of the interstices and channels. To meet the goal of making the calculations automatic and robust, we introduce the distance from the interstice/channel to the surface of the nearest framework atom as the weights for the evaluation (see S2 of the Supplementary Information for details). After that, the accessible subspace of the void space can be easily determined by comparing the interstitial/bottleneck size in the network with the statistical values for mobile ions in different coordination environments (see subsection “Ionic transport descriptors”).

**Fig. 3:**
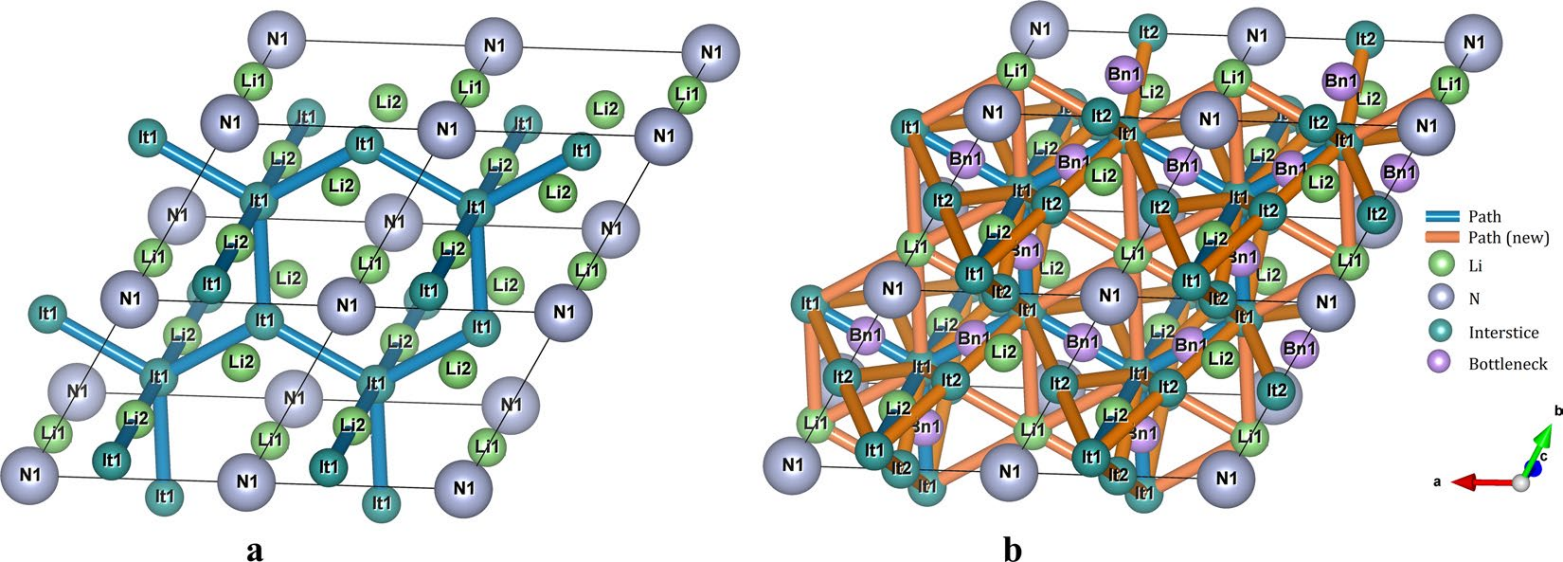
The Voronoi network (**a**) and interstitial network (**b**) obtained from the N^3−^ packing in α-Li_3_N (icsd_156890). The Li1 site of α-Li_3_N cannot be identified by Voronoi network, so that the pathways Li1-Li2 and Li1-Li1 cannot be obtained. In contrast, our interstitial network, which includes centers of Voronoi polyhedra faces, identifies all Li sites of α-Li_3_N and thus migration pathways.

The resulting weighted Voronoi network *WVN = *(*V*, *E*) is comprising a set *V* of interstices together with a set *E* of channel segments, which are 2-element subsets of *V*. Only with all the sites of mobile ions matched with the interstices that have been included in the *WVN*, can we get all migration pathways between them. However, in some structures, we cannot find the interstices contained in the network that correspond to the lattice sites of target ions. For example, the lattice positions of Li1 and Li2 in α-Li_3_N do not coincide with any Voronoi vertices. Li1 locates at the center of the Voronoi face, and Li2 occupies the bottleneck site of the channel connection two adjacent interstices (Fig. 3(a)). This is an example of the situation when a 3D Voronoi polyhedron degenerates into a 2D polygon, which, similarly to channel size, should be neither too wide nor too narrow^26,36,64,65^. Therefore, we extend the set *V* to include the interstices that coincide with the center of Voronoi faces, and place the connections of the centers and the vertices of the Voronoi faces into the set *E*. The acquired new interstitial network (Fig. 3(b)) is characterized by the position and size of interstices, channels between interstices, and bottlenecks of the channels which are used to determine possible migration pathways.

### Ionic transport descriptors

A descriptor, an effective input data representation for material characteristics, is critical to study structure–property relationships^66^. Some efforts have been made in the development of material descriptors based on Voronoi decomposition. With the help of Voronoi node and its coordinating host-lattice atoms, Kahle *et al*.^60^ introduced a landmark vector to perform a site analysis of molecular dynamics trajectories for ionic diffusion analysis. Based on Voronoi-tessellation real feature values and atomic property data, Jalem *et al*.^67^ proposed a general representation scheme for crystalline solids, which showed good predictive power and generalization performance in the density functional theory based calculation of material properties. Yang *et al*.^68^ defined a function to quantify structure similarity and predict populated crystal sites using both Voronoi cell and chemical composition. In these schemes, descriptors are all based on the basic geometric/topological features contained in the Voronoi cell and the problem-specific domain knowledge (chemical/atomic information). In this work, instead we focus on more general geometrical/topological descriptors that are frequently used in ionic transport.

**Fig. 4:**
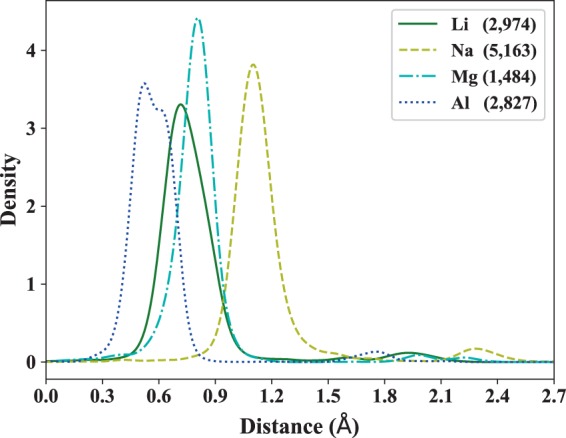
The density plot of the distances from lattice sites of mobile ions (Li^+^, Na^+^, Mg^2+^, and Al^3+^) to the surfaces of their nearest framework ions in 12,448 coordination environments. The density estimate plots of the minimal distances under different coordination environments (distinguished by coordination number) for Li^+^, Na^+^, Mg^2+^, and Al^3+^ are shown in S3 (a), (b), (c), and (d) of the Supplementary Information, respectively. Except that the maxima at the higher distances correspond to the next coordination shell, most of the minimum distances are distributed within a well-defined range. The double peeks of Al^3+^ are caused by inconsistent data distribution. The density estimate plot of Al^3+^ is obtained from the minimum distances calculated under CN = 4 (1,478 samples), CN = 5 (66 samples), and CN = 6 coordination (1,283 samples), but the minimum distances corresponding to CN = 4, CN = 5, and CN = 6 are sparsely distributed.

For the beginning, the criteria for ion accessibility need to be determined. To quantify such criteria, distances from the sites of mobile ions sublattice to their nearest immobile framework ions surface were calculated in 12,448 coordination environments. A kernel density estimate plot (Fig. 4, S3 of the Supplementary Information) is formed by computing a continuous probability distribution estimate (using Gaussian kernels with 1000 equally evaluation points) of the minimal distances. The calculated distances are distributed within a well-defined range, so the lower threshold (*T*_*l*_) and upper threshold (*T*_*u*_) of ion migration can be easily determined. In order to cover as many samples as possible and avoid excessively long distances with high coordination numbers, the thresholds are obtained from about 90% of the data (Table 1) and then used to determine the mobile ions accessibility of the voids in Li-, Na-, Mg- and Al-containing compounds. If the size *r* of the interstice or bottleneck satisfies *T*_*l*_ ≤ *r* ≤ *T*_*u*_, it is considered to be accessible.

**Table 1:** Lower threshold (*T*_*l*_) and upper threshold (*T*_*u*_) used to determine the ionic accessibility (for Li^+^, Na^+^, Mg^2+^, and Al^3+^) of the interstice and bottleneck.

	*T*_*l*_ (Å)	*T*_*u*_ (Å)
Li^+^	0.5267	0.9857
Na^+^	0.9295	1.3961
Mg^2+^	0.5513	1.0081
Al^3+^	0.3447	0.7307

Based on the *T*_*l*_ and *T*_*u*_, the basic geometrical/topological descriptors for transport channel can be acquired, including the connected transport channel (*TC*), the interstitial network dimension (*IND*), the radius of largest ion that can freely pass through the void space (*R*_*T*_), the critical radii for main crystallographic directions *a* (*R*_*Ta*_), *b* (*R*_*Tb*_) and *c* (*R*_*Tc*_).

**Fig. 5:**
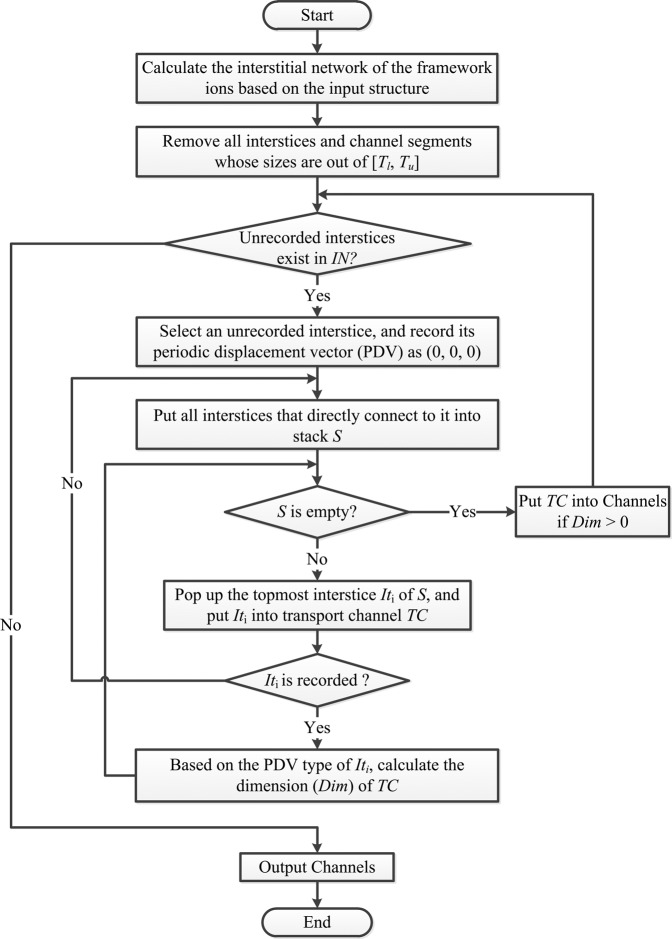
Workflow of the channel analysis algorithm.

***TC***: The transport channel is formed by connected interstices and channel segments whose size is within [*T*_*l*_, *T*_*u*_]. The *TC* characterizes the continuous subspace of the void space accessible by mobile ions^12–14^ and is useful for identifying the approximate minimum energy path of ion migration^29,69^ Each interstice that belongs to the transport channel should have the pathways to its periodic images in adjacent cells. The algorithm^22^ (shown in Fig. 5) for calculating the transport channel is described as following:

1. Calculate the interstitial network of the framework ions based on the input structure;
2. Remove all interstices and channel segments which sizes are out of [*T*_*l*_, *T*_*u*_];
3. Select an unrecorded interstice, then record its periodic displacement vector as (0, 0, 0), and push all interstices that directly connected to the interstice into a stack. When the stack is not empty, remove the topmost interstice and performs following analysis:
  - If the interstice has not been recorded, record its periodic displacement vector and push all directly connected interstices into the stack;
  - If the interstice has been recorded with other periodic displacement vectors, all its connected interstices constitute a transport channel;
4. Repeat step (3) until all interstices have been recorded.

It should be noted that there may be more than one transport channel in a structure, and all of them form a transport network.

***R***_***T***_: The size of restricting interstice or bottleneck in the interstitial network. *R*_*T*_, characterizes the radius of largest ion that can freely pass through the void space. This descriptor is analogous to the radius of largest free sphere (RLFS), a currently widely used description of the pore geometry^22,70–72^ in crystalline porous materials. The RLFS refers to the maximum size of a spherical probe that can travel through the void space in a structure by at least one periodic lattice translation, and can be calculated for three crystallographic directions. Taking the same approach, we define *R*_*T*_ as the size of restricting interstice or bottleneck in the interstitial network to characterize the ion transport. The *R*_*T*_ for three crystallographic directions *a* (*R*_*Ta*_), *b* (*R*_*Tb*_) and *c* (*R*_*Tc*_) are also obtained, and *R*_*T*_* = *max{*R*_*Ta*_, *R*_*Tb*_, *R*_*Tc*_}.

***IND***: The interstitial network dimension. The *IND* provides the information about the dimensionality of the network that mobile ions can diffuse through in the void space^12–14^. The interstitial network may be one- (1D), two- (2D) or three-dimensional (3D) or otherwise, zero dimensional (0D) if no infinite channels are found. The value of *IND* is determined from *R*_*Ta*_, *R*_*Tb*_ and *R*_*Tc*_ with *T*_*l*_. For example, if *R*_*Ta*_ larger than (or equal to) the *T*_*l*_, the interstitial network will be called connected in the *a* direction, and if only one crystallographic direction connected, the system will be called 1D.

## Results and Discussions

To support our work, 31,499 Crystallographic Information Files (CIFs)^73,74^ for Li, Na, Mg and Al containing compositions were extracted from the Inorganic Crystal Structure Database (ICSD; release 2010/2)^75^. For these compounds, the repairable format errors (such as inconsistent bracket and excessive blank lines) were corrected at first. The data cleaning procedure (e.g., removal of elements, alloys, and entries with hydrogen and H isotopes, or errors in the atomic sites), reduced the set to 17,206 entries. After removing entries with duplicated and undetermined atom sites, 6,955 entries having Li (1,920 items), Na (2,841 items), Mg (1,125 items) and Al (1,743 items) were selected with fully occupied metal sites whose positions are accurately determined. The detailed process of data cleaning is described in S4 of the Supplementary Information.

### Methodology validation

**Fig. 6:**
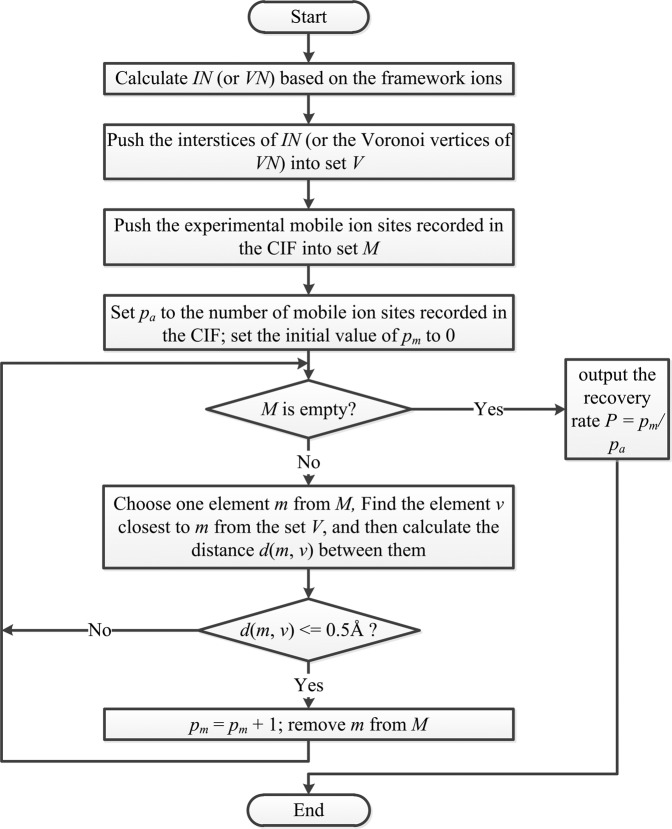
Workflow of the recovery rate calculation.

Since one of the main purposes of our tool is to identify pathways for further FP-NEB calculations, the prerequisite for the CAVD analysis is that all experimental lattice sites of mobile ions are matched to the interstitial network. As a first step of the methodology validation, we assess the ability of the calculations to recover the positions of the mobile ions for all 6,955 compounds using only framework structure information. For each compound, we remove mobile ions, then calculate the interstitial network (*IN*) using our approach as described in section “Methods”, and standard Voronoi network (*VN*) (for comparison) for the framework and accumulate the possible mobile ions sites (the interstices of *IN*, or the Voronoi vertices of *VN*) in a set *V*_*IN*_ and *V*_*VN*_, respectively. The experimental sites are stored in set *M*. The recovery rate for every compounds can be determined by the algorithm^68^ (Fig. 6) described as following:

1. Check the recovery state *p*_*m*_ of each mobile ion site (*m*) based on the distance between *m* and the nearest element (*v*) in *V*_*IN*_ (or *V*_*VN*_): if *d*(*m*, *v*) < = 0.5 Å, *p*_*m*_ = 1; otherwise, *p*_*m*_ = 0. (The threshold 0.5 Å is adopted from the Yang’s work^68^).
2. If size of set *M* is *n*, the recovery rate *P* of the compound satisfies

4 $$P=\frac{\mathop{\sum }\limits_{m=1}^{n}{p}_{m}}{n}.$$

We classify the recovery rate of every compound into three types: complete recovery (*P* = 1), partial recovery (0 < *P* < 1), and a miss (*P* = 0). The recovery rates of all 6,955 compounds for *IN* and *VN* are presented in Table 2. It can be clearly seen that our model performs well in characterizing mobile ion sites with a high rate of complete recovery and very low miss rate. The performance of the standard Voronoi network is slightly inferior. Upon investigation, we found that mobile ions in these compounds lie on the Voronoi faces and thus cannot be matched by any Voronoi vertices. In contrast, in our model we consider the centers of the Voronoi faces, the positions closest to an atom on the Voronoi edges as well as the Voronoi vertices as possible mobile ions locations. Therefore, our model has a higher recovery rate than the standard model. We also notice that in ~1% of the 6,955 compounds which were only partially recovered by our model (Table 2), some of the distances *d*(*m*, *v*) are just slightly larger than the threshold 0.5 Å. If the threshold is increased, those compounds are also completely recovered (S5 of the Supplementary Information). All of these discussions clearly demonstrate that our method successfully identifies mobile ion sites and the channels between them.

**Table 2:** The rate of Li-, Na-, Mg- and Al-containing compounds under different recovery rate types. Listed are the mobile ion, the total number of the analyzed CIFs containing mobile ion, three types of recovery rate calculated from standard Voronoi network and interstitial network, respectively.

Mobile ion	Total	*P* = 1		0 < *P* < 1		*P* = 0	
		*VN*	*IN*	*VN*	*IN*	*VN*	*IN*
Li^+^	1920	90.21%	98.18%	3.80%	1.09%	5.99%	0.73%
Na^+^	2841	92.36%	98.52%	4.22%	1.13%	3.42%	0.35%
Mg^2+^	1125	97.96%	99.30%	0.88%	0.08%	1.16%	0.62%
Al^3+^	1743	97.99%	99.66%	0.57%	0.23%	1.43%	0.11%

### Calculated results

To further validate the developed code, we calculated the ionic transport descriptors for selected 6,995 compounds as shown in the supplementary files (in figshare). The calculated transport channel for each CIF is provided by *.vesta files and *.net files (archived in Channels.zip). The remaining numeric descriptors are recorded in Descriptors.xlsx, and a short description of all data is given in S6 of the Supplementary Information.

The results, including 17 well known ionic conductors, including Li-nitride, NASICON, Garnet, LIPHOS, perovskite, and β-Al_2_O_3_, are consistent with previous reports (shown in Table 3). In order to demonstrate the features of our method, four well-studied materials are discussed in detail. Na_4_Zr_2_(SiO_4_)_3_ and Li_7_La_3_Zr_2_O_12_ with 3D transport channel are presented in this section, LiFePO_4_ with 1D channel and Na_2_O(Al_2_O_3_)_11_ with 2D channel are shown in the S7 of the Supplementary Information.

**Table 3:** Calculated descriptor values of 17 well-known ionic conductors.

Chemical formula	Space group	*R*_*T*_ (Å)	*R*_*Ta*_ (Å)	*R*_*Tb*_ (Å)	*R*_*Tc*_ (Å)	*IND*	ICSD	Dim in Ref.
**Li-nitride**								
α-Li_3_N	*P*6/*mmm*	1. 347	1.347	1.347	0.7919	3D^§^	660252	2D^29^
**NASICON**								
LiTi_2_(PO_4_)_3_	*R*-3*c*	0.7574	0.7574	0.7574	0.7574	3D	95979	3D^29^
LiGe_2_(PO_4_)_3_	*R*-3*c*	0.7227	0.7227	0.7227	0.7227	3D	69763	3D^29^
Na_4_Zr_2_(SiO_4_)_3_	*R*-3*c*	0.9645	0.9645	0.9645	0.9645	3D	38055	3D^12^
**Garnet**								
Li_7_La_3_Zr_2_O_12_	*I*4_1_/*acd*	0.5631	0.5595	0.5595	0.5631	3D	246817	3D^76^
**LIPHOS**								
Li_3_Sc_2_(PO_4_)_3_	*P*112_1_/*n*	0.6904	0.6826	0.6826	0.6904	3D	62301	3D^12^
Li_3_Fe_2_(PO_4_)_3_	*R*-3*c*	1.3483	1.3483	0.6985	0.6985	3D	95973	3D^12^
**Perovskite**								
LiLaTiO_4_	*P*4/*nmm*	0.8766	0.8766	0. 8158	0.6461	3D	81857	3D^3^
**β-Al**_**2**_**O**_**3**_								
Na_2_O(Al_2_O_3_)_11_	*P*6_3_/*mmc*	1.2851	1.2851	1.2851	0.3315	2D	60635	2D^77^
**Others**								
LiF	*Fm*-3*m*	0.4634	0.4634	0.4634	0.4634	0D	44272	0D^26^
Li_2_CO_3_	*C*12/c1	0.6942	0.6942	0.6942	0.6390	3D^§^	16713	2D^26^
**Electrodes**								
LiFePO_4_	*Pnma*	0.6557	0.4373	0.6557	0.4788	1D	56291	1D^29^
LiCoO_2_	*R*-3*m*	0.4439	0.4439	0.4439	0.3646	0D^§^	98270	2D^15^
Li_2_TiO_3_	*C*12/c1	0.8501	0.8501	0.5612	0.5526	3D	15150	3D^29^
LiMn_2_O_4_	*Fddd*	0.5861	0.5861	0.5861	0.5861	3D	54700	3D^29^
Na_2_V_3_O_7_	*P*31*c*	1.6674	1.6674	1.6674	1.1365	3D	88780	3D^69^
Na_2_CoP_2_O_7_	*P*4_2_/*mmm*	1.0342	1.0342	1.0342	0.7863	3D^§^	50785	2D^69^

^§^*R*_*Tc*_ ≪ *R*_*Ta*_ = *R*_*Tb*_ effectively makes the material as 2D conductor in agreement with reports in^15,78,79^.

The first material is a NASICON (Na superionic conductor) type compound Na_4_Zr_2_(SiO_4_)_3_^80^. It is a good ion conductor due to the network of wide channels in the three-dimensional rhombohedral *R-3c* framework of corner sharing ZrO_6_ octahedra and SiO_4_ tetrahedra (Fig)^80–82^. Mobile Na ions are distributed over the two types of interstices: Na1 (in an octahedral oxygen environment at the intersection of three transport channels) and Na2 (in an 8–10 oxygen environment at each bend of the conduction channels) and the transport involves the Na1-Na2-Na1 migration.

**Fig. 7:**
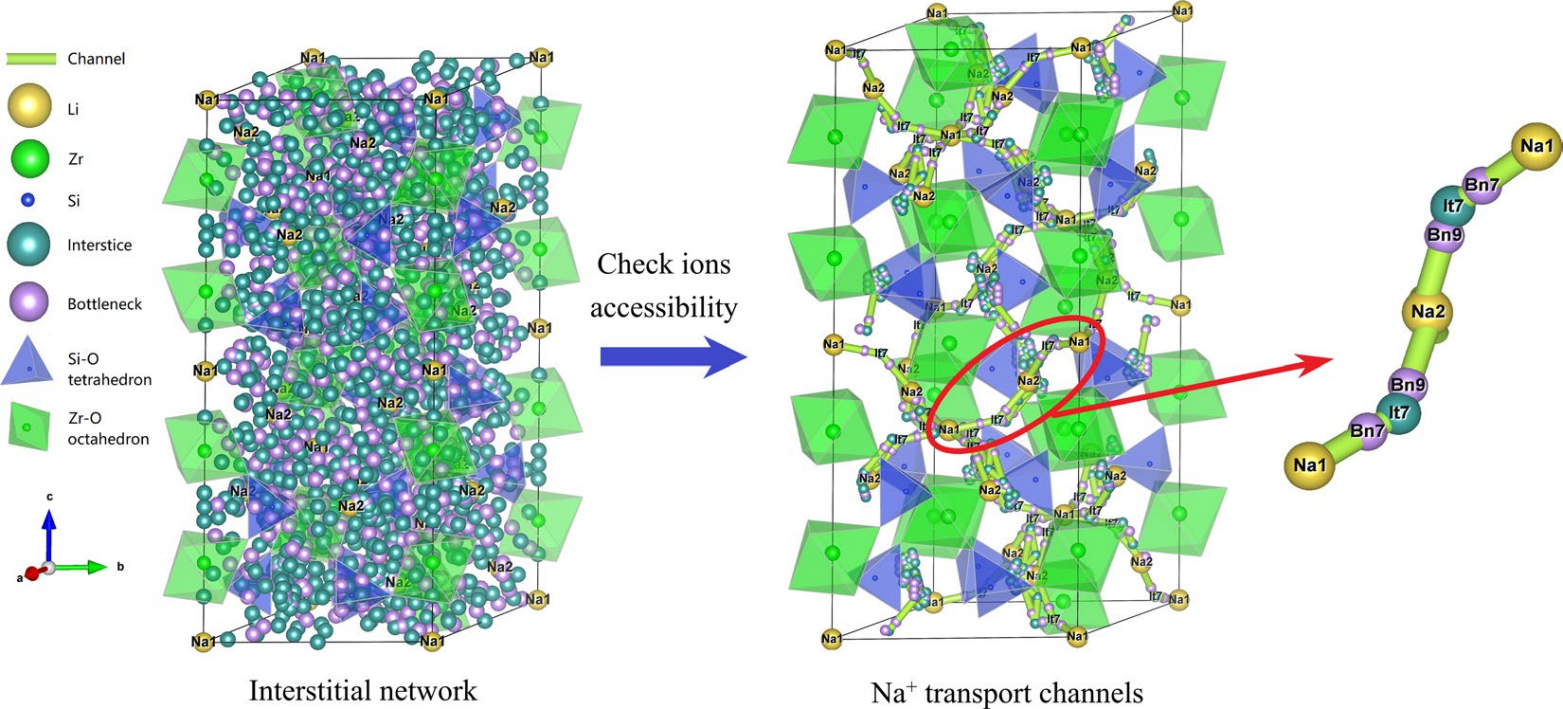
The 3D transport channel for Na^+^ in Na_4_Zr_2_(SiO_4_)_3_ (icsd_38055). With the help of step (2), the Na^+^ transport channel with 402 interstices is obtained from the interstitial network with 1,182 interstices. These 402 interstices are then merged with symmetry equivalent (step (3)), and 13 symmetrically distinct interstices are obtained. The symmetrically distinct interstice and bottleneck are labeled as It and Bn, respectively. All interstices and channel segments in channel (see VestaFiles/icsd_38055.vesta in figshare) can be accessed by Na^+^, but some of them are omitted in the figure for clarity.

Before applying our method to Na_4_Zr_2_(SiO_4_)_3_, all Na ions are removed from the structure and the following steps are carried out to locate the transport channels:

1. Calculate the interstitial network based on the framework structure;
2. Calculate the connected transport channels by applying the geometric transport analysis algorithm to the interstitial network;
3. Simplify the transport channels by identifying crystallographically equivalent interstices;
4. Identify the experimental sites, and the pathways between them form simplified channels.

All of the steps are done by the code automatically and no manual processing of the interstitial network is done by hand. The procedure yielded a 3D Na^+^ network consisting of 13 symmetrically distinct interstices and 24 channel segments between the interstices (see Table S4 of the Supplementary Information). In this network, the centers of It6 and It9 coincide with the experimental equilibrium sites Na1 and Na2, respectively. The paths between Na1 and nearest Na1 is Na1(It6)-It7-Na2(It9)-It7-Na1(It6) (Fig. 7), which is consistent with previous reports^80,81^. Moreover, an interface is provided to automatically generate a series of images along the migration path and the POSCAR files corresponding to images for further FP-NEB analysis.

**Fig. 8:**
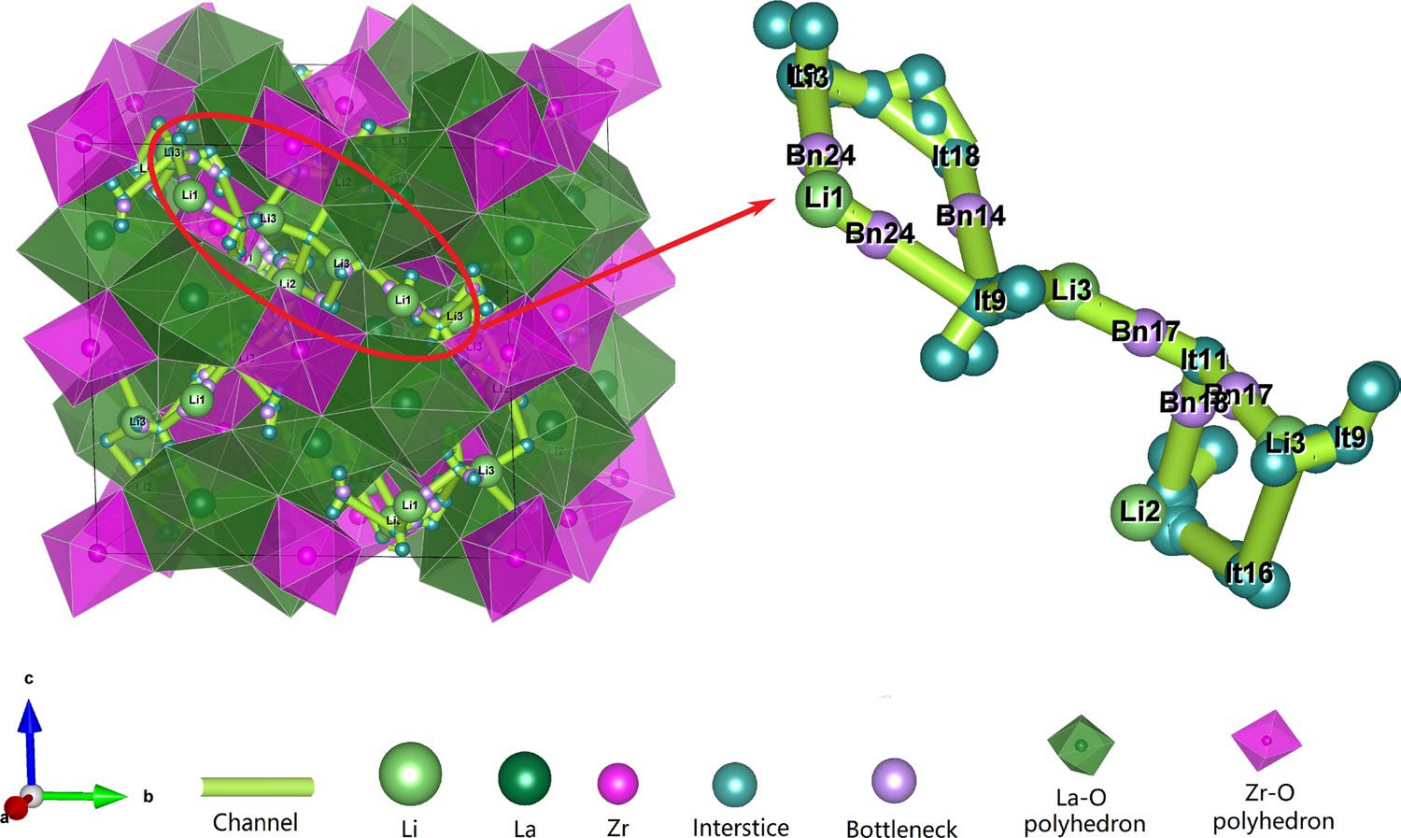
The 3D Li^+^ transport channel in Li_7_La_3_Zr_2_O_12_ (icsd_246817). All of the reported migration paths between Li1, Li2 and Li3 are included in the calculated channel (see VestaFiles/icsd_246817.vesta in figshare). There are five interstitial clusters are formed by It10-It12-It17, It7-It18, It6-It16, It9-It15 and It1-It2, some of them are omitted in the figure for clarity.

The garnet-type compounds are promising candidates as solid electrolytes for solid-state batteries in view of the high ionic conductivity, wide electrochemical window and good chemical stability with respect to metallic lithium anode^4,6,76,83^. Among the garnet family, Li_7_La_3_Zr_2_O_12_ (LLZO) has two phases: high-temperature cubic phase (cubic-LLZO) and low-temperature tetragonal phase (tetragonal-LLZO). In the tetragonal-LLZO, Li ions occupy three different sites: the tetrahedral sites (Li1), the octahedral sites (Li2), and the tetrahedral sites (Li3). The 3D transport network in tetragonal-LLZO consists of face-shared tetrahedra and octahedra. After analysis by our method, we reproduce the same 3D transport channel consisting of 24 symmetrically distinct interstices (see Table S5 in Supplementary Information). In the channel (Fig. 8), It19, It10, and It13 coincide with Li1, Li2, and Li3, respectively. The calculated channel between Li3 and Li3, which are connected through three paths: Li3(It13)-Bn24-Li1(It19)-Bn24- Li3(It13), Li3(It13)-It18-Bn14-Li3(It13), and Li3(It13)-Bn17-It11-Bn17-Li3(It13), are selected as example.

**Fig. 9:**
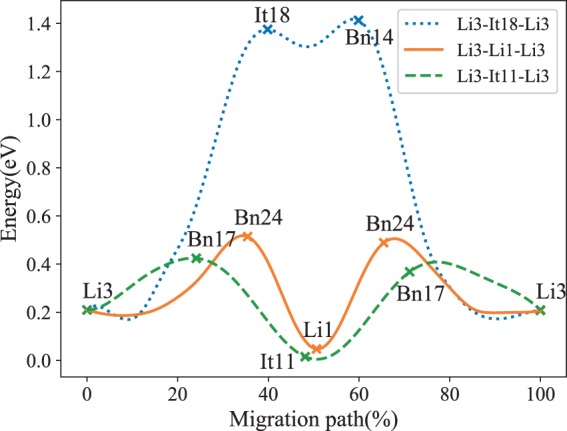
The energy profiles of the paths between neighboring Li3 sites in Li_7_La_3_Zr_2_O_12_. The bottlenecks and interstices in Li3-It11-Li3 and Li3-Li1-Li3 coincide with the peak and valley of the energy profiles.

Based on the results of these tests, we are confident that our model has the ability to identify all possible channels between the mobile ions. The results can be further combined with BVSE calculations to identify the minimum energy paths as illustrated by the BVSE energy profiles of the pathways Li3-Li3 in Li_7_La_3_Zr_2_O_12_ performed using our joint calculation program^31^. As expected, we find the interstices in paths Li3-Li1-Li3 and Li3-It11-Li3 are distributed at the valley of energy profile, and the bottlenecks coincide with the peak (Fig. 9). As the abnormal path Li3-It18-Li3 in the energy profile, it seems to be hard to transport because of the high energy barrier. Along this path, both It18 and Bn14 are located at high energy sites. After we analyzed the specific locations of the interstices, we found that It18 coincides with the center of a new tetrahedron sharing edge with the tetrahedron occupied by Li1, and the centers of Bn18 are located on the face of the new polyhedron. In addition, the low energy paths Li3-Li1-Li3 and Li3-It11-Li3 are observed to be similar, where Li1 and It11 are also located in the Li-O tetrahedron. This small difference is caused by the unequal lattice length of the tetragonal-LLZO.

We can summarize that our tool, as the other Voronoi decomposition based codes, offers a very quick way to assess any material as a potential ionic conductor based on the crystal structure information. The suitable geometry of the framework is not a sufficient condition for a very good percolation-type ionic conductivity, but it is definitely a necessary condition and the method can be used to filter out the structures which do not favour ionic transport. Another useful application of the method is that it allows identifying the position of all the segments of the conduction channels that can be used as input for *ab initio* nudged elastic band calculations.

As all the other Voronoi tessellation codes, our tool has some limitations. The mobile ion is needed to be specified before applying CAVD, but this will become difficult when compound contains two or more kinds of potential mobile ions. Since the radical Voronoi decomposition is sensitive to changes in local structure^54^, the interstitial clusters constructed by Voronoi cell make it difficult to identify of the sites of mobile ions and the symmetry of the interstices. The procedure that simplify the network by merging all interstitial clusters as their geometric center is in progress and will be published elsewhere. Furthermore, ion radius is only one of the factors affecting the mobility of ions in ionic conductors^84^, improvement of the tool with additional criteria, such as formation energies, can make the results more precise and robust; defining a simple, measurable parameter to describe the distribution of framework ions, and establishing the relationship between the parameter and ionic transport will make CAVD-based structure prediction possible^85^.

## Conclusion

We developed the Crystal structure Analysis by Voronoi Decomposition (CAVD) tool to characterize the void space of crystal structures as interstitial network by the radical Voronoi decomposition. The obtained interstitial network can be used to calculate crystal structure characteristics relevant for ionic transport properties. We validated our code by predicting the lattice sites of mobile ions in 6,955 compounds extracted from the ICSD with the success rate > 98%. The detailed study of the migration channels in Na_4_Zr_2_(SiO_4_)_3_ and Li_7_La_3_Zr_2_O_12_ also confirmed the validity of our method. In addition, 6,955 descriptor data that can be further studied, e.g. material machine learning, structure classification, and structure–property relationships study, are shared. CAVD can be used not only to identify the position of mobile ion sites and conduction channels and quantify the accessible void space, but also to provide insights for BVSE or FP-NEB calculations for the newly established high-throughput screening platform for battery materials (https://www.bmaterials.cn)^30^.

## Supplementary information

Supplementary Information

## Data Availability

The data (all files mentioned in the main text and the Supplementary Information) that support the findings of this study are available from the figshare^86^.

## Code availability

Source codes of CAVD are freely accessed in the repository: https://gitee.com/shuhebing/cavd.
