# Supplementary material for: CAVD, towards better characterization of void space for ionic transport analysis
Source: Sci Data. 2020 May 22;7:153. doi: 10.1038/s41597-020-0491-x (PMC7244509; doi:10.1038/s41597-020-0491-x)
Supplement: Supplementary file 1 — Supplementary Information [file 41597_2020_491_MOESM1_ESM.docx]

CAVD, Towards better characterization of void space for ionic transport analysis

Bing He^1^, Anjiang Ye^1^, Shuting Chi^1^, Penghui Mi^1^, Yunbing Ran^2^, Liwen Zhang^2^, Xinxin Zou^1^, Bowei Pu^2^, Qian Zhao^3^, Zheyi Zou^2^, Da Wang^2^, Wenqing Zhang^4^, Jingtai Zhao^5^, Maxim Avdeev^6,7^, Siqi Shi^2,3,*^

^1^*School of Computer Engineering and Science, Shanghai University, Shanghai 200444, China*

^2^*State Key Laboratory of Advanced Special Steel, School of Materials Science and Engineering,*

*Shanghai University, Shanghai 200444, China*

^3^*Materials Genome Institute, Shanghai University, Shanghai 200444, China*

^4^*Department of Physics and Shenzhen Institute for Quantum Science & Technology,*

*Southern University of Science and Technology, Shenzhen, 518055, China*

^5^*School of Materials Science and Engineering, Guilin University of Electronic Technology, Guilin 541004, China*

^6^*Australian Nuclear Science and Technology Organisation, Locked Bag 2001, Kirrawee DC NSW 2232, Australia*

^7^*School of Chemistry, The University of Sydney, Sydney 2006, Australia*

^*^corresponding author: Siqi Shi (sqshi@shu.edu.cn)

## Analysis of standard Voronoi decomposition, radical Voronoi decomposition and Voronoi S decomposition

As shown in equation (1), (2) and (3), the interstice and bottleneck of our model depend on the radius *r* of the Voronoi generators and the distance *d* between them. In Fig. 2, *r*_A_ = *r*_B_ = *r*_C_ = *r*_D_ = 0.4Å, *r*_E_ = 1Å, *d*(A, B) = *d*(B, C) = *d*(C, D) = *d*(D, A) = 3.2Å, *d*(A, E) = *d*(B, E) = *d*(C, E) = *d*(D, E) = 2.26Å. O_V_, O_S_ and O_R_ are the vertices of the standard Voronoi cell, Voronoi S cell and radical Voronoi cell, respectively. In the local environment formed by circle C, D and E, the center of local largest void coincides with O_S_, and the deviation of O_R_ from O_S_ (0.5175Å) is less than the deviation of O_V_ from O_S_ (0.78Å). The sizes of the interstices corresponding to O_R_ and O_V_ are 0.8625Å and 0.6Å, respectively. The sizes of the bottleneck on O_V_O_V_, O_S_O_S_ and O_R_O_R_ are 0.1314Å, 0.4314Å (the real bottleneck size) and 0.317Å, respectively. Based on the examples like this, we can conclude that the radical Voronoi decomposition provides more adequate description of the void space than standard Voronoi decomposition for a structure with atoms of unequal radii.

Actually, the deviations of interstice and bottleneck are caused by different boundary definitions. In 2D space, the boundaries of standard Voronoi cell and radical Voronoi cell are straight line, but the boundary of Voronoi S cell is a hyperbolic curve. The intersection of the boundary with the line connecting the centers of adjacent generators corresponds to the center of bottleneck. Let generator P with coordinates (0, 0) and radius *r*_P_, generator Q with coordinates (*d*, 0) and radius *r*_Q_. The boundaries (Figure S1) formed by P and Q based on standard Voronoi decomposition, radical Voronoi decomposition, and Voronoi S decomposition are labeled as Std, Rad, and S, respectively. The coordinates of L are ($\frac{d}{2}$, 0) and the size of the bottleneck (*B*_Std_) corresponding to L is $\frac{d}{2}-r_{\text{P}}$. The coordinates of M are ($\frac{d^{2}+r_{\text{P}}^{2}-r_{\text{Q}}^{2}}{2d}$, 0), and the size of the bottleneck (*B*_Rad_) corresponding to M is $\frac{d^{2}+r_{\text{P}}^{2}-r_{\text{Q}}^{2}}{2d}-r_{\text{P}}$. The coordinates of N are ($\frac{d+r_{\text{P}}-r_{\text{Q}}}{2}$, 0), and the size of the bottleneck (*B*_S_) corresponding to N is $\frac{d+r_{\text{P}}-r_{\text{Q}}}{2}-r_{\text{P}}$. *B*_S_ is the most accurate size of the bottleneck in the local environment. $\frac{\text{PL}}{\text{PN}}=\frac{d}{d+r_{\text{P}}-r_{\text{Q}}}$, $\frac{\text{PM}}{\text{PN}}=\frac{d^{2}+r_{\text{P}}^{2}-r_{\text{Q}}^{2}}{d^{2}+d(r_{\text{P}}-r_{\text{Q}})}$, therefore, the closer *r*_P_ is to *r*_Q_, the closer *B*_Std_ is to *B*_S_, and the closer (*r*_P_ + *r*_Q_) is to *d* or *r*_P_ is to *r*_Q_, the closer *B*_Rad_ is to *B*_S_. Moreover, when *r*_P_ = *r*_Q_, *B*_Std_ = *B*_Rad_ = *B*_S_.


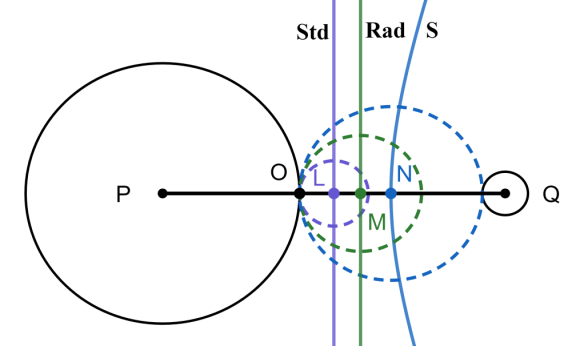


**Figure S1.** The boundaries of standard Voronoi cell (purple lines), radical Voronoi cell (green lines) and Voronoi S cell (blue lines).

## The calculations of interstice and bottleneck

The interstice *v* formed by framework atoms *a*_1_, *a*_2_, *a*_3_ and *a*_4_ (with radii *r_1_*, *r_2_*, *r_3_* and *r_4_*, respectively; usually four in 3D space) satisfies the following data structure (Figure S2):

1. position: *v* (*x, y, z*);
2. size: min{*d*(*v*, *a*_1_) – *r_1_*, *d*(*v*, *a*_2_) – *r_2_*, *d*(*v*, *a*_3_) – *r_3_*, *d*(*v*, *a*_4_) – *r_4_*}.

The distance between the channel segment *v_i_v_j_* (formed by interstices *v_i_* and *v_j_*) and its surrounding framework atoms can be calculated by a vector algorithm. Let one of the framework atoms is *a_k_*, a vector with beginning point *v_i_* and end point *a_k_* be denoted by ***u***, and a vector with beginning point *v_i_* and end point *v_j_* be denoted by ***w***. The vector projection of ***u*** on ***w*** satisfies:

$proj_{\mathbf{w}}\mathbf{u}=\frac{\mathbf{u}\cdot\mathbf{w}}{\left|\mathbf{w}\right|^{2}}\mathbf{w}.$ (S1)

The value of $\frac{\mathbf{u}\cdot\mathbf{w}}{\left|\mathbf{w}\right|^{2}}$ (denoted as *p_k_*) can be used to reflect the relative position of *a_k_* and *v_i_v_j_.* Let the point closest to *a_k_* on *v_i_v_j_* be *q_k_*, the vector ***v*** with beginning point *v_i_* and end point *q_k_* satisfies:

$\mathbf{v}=p_{k}\mathbf{w},p_{k}=\left\{\begin{matrix} 0 \\ p_{k} \\ 1 \end{matrix}\right.\begin{matrix} p_{k}\leq0 \\ 0<p_{k}\leq1 \\ p_{k}>1 \end{matrix}.$ (S2)

With the help of equation (S2), the coordinates of *q_k_* and the distance *d*(*q_k_, a_k_*) between *q_k_* and *a_k_* can be easily obtained. If *v_i_v_j_* is surrounded by framework atoms *a*_1_, *a*_2_ and *a*_3_ (with radii *r_1_*, *r_2_* and *r_3_*, respectively; usually three in 3D space), the data structure of *v_i_v_j_* can be summarized as following (see Figure S2):

1. connection: (*v_i_*, *v_j_*);
2. bottleneck size: min{*d*(*q_1_*, *a*_1_) – *r_1_*, *d*(*q_2_*, *a*_2_) – *r_2_*, *d*(*q_3_*, *a*_3_) – *r_3_*};
3. bottleneck position: *q*(*x, y, z*).


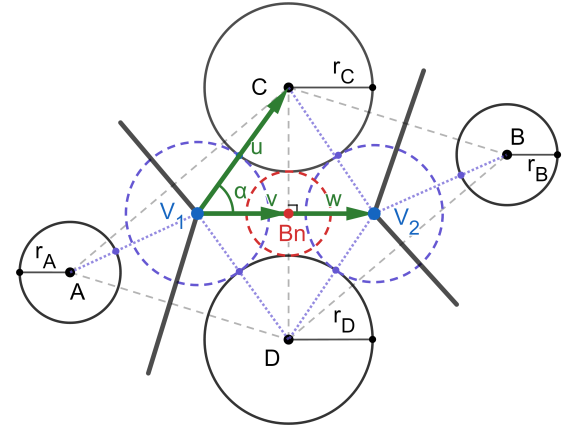


**Figure S2.** The interstice and bottleneck in 2D space. The interstice (presented as purple dotted circles) V_1_ is formed by framework atoms A, C and D; the interstice V_2_ is formed by framework atoms B, C and D; the bottleneck of channel segment V_1_V_2_ is Bn (presented as red dotted circle). The distances between V_1_ and its constructed framework atoms are *d*(V_1_, A), *d*(V_1_, C) and *d*(V_1_, D). The size of V_1_ is min{*d*(V_1_, A) – r_A_, *d*(V_1_, C) – r_C_, *d*(V_1_, D) – r_D_}. The distances between V_1_V_2_ and its surrounding framework atoms are *d*(Bn, C) and *d*(Bn, D). The size of Bn is min{*d*(Bn, C) – r_C_, *d*(Bn, D) – r_D_}. Both the coordinates of Bn and the distances can be obtained by equation (S2).

## The estimate density plots of the minimal distances

| 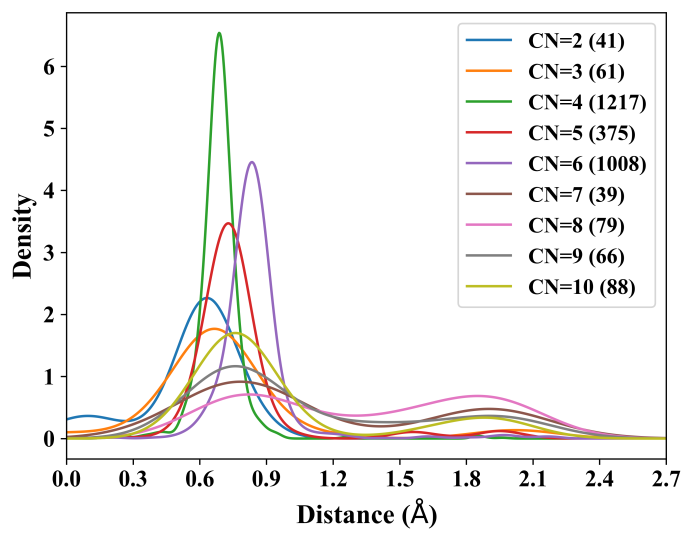 | 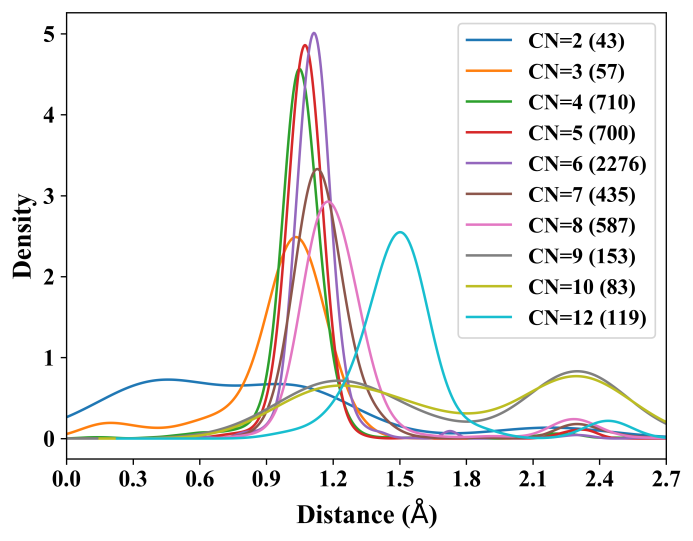 |
| --- | --- |
| (a) | (b) |
| 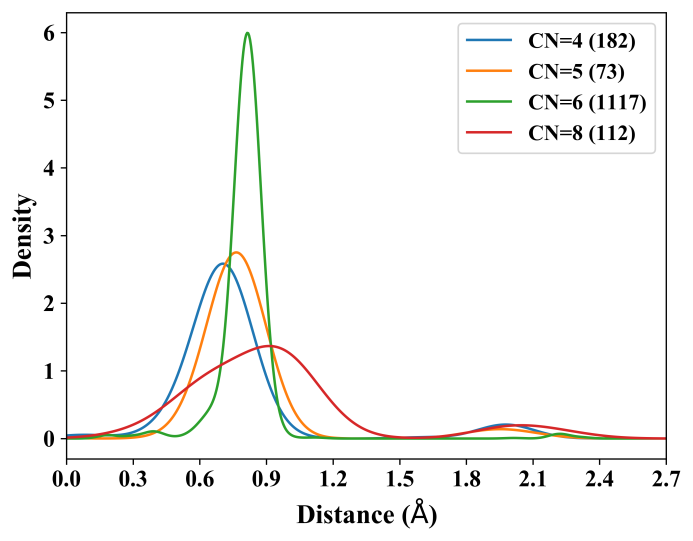 | 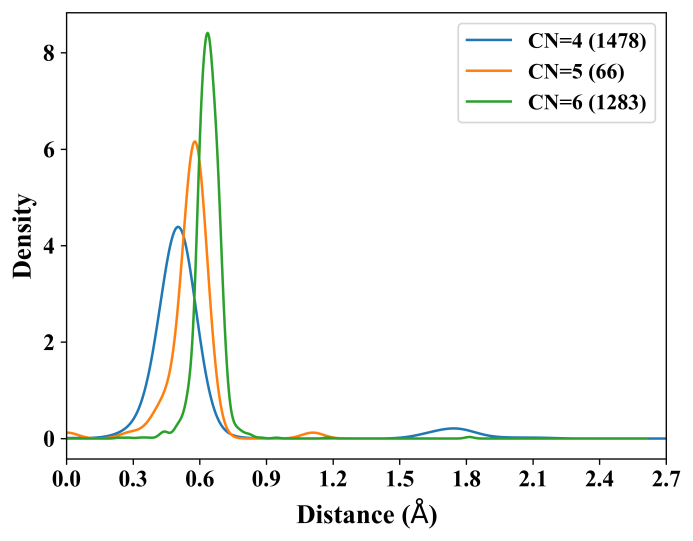 |
| (c) | (d) |

**Figure S3.** The estimate density plots of the minimal distances from lattice sites of Li^+^ (**a**), Na^+^ (**b**), Mg^2+^ (**c**) Al^3+^ (**d**) to the surfaces of the nearest framework ions under different coordination environments.

## Data cleaning

As a basis of our work, 31,499 Crystallographic Information Files (CIFs) for Li, Na, Mg and Al containing compositions were extracted from the Inorganic Crystal Structure Database (ICSD; release 2010/2).^1^ At first, we correct some repairable format errors, including:

1. inconsistent bracket (for example, appeared in icsd_415733, icsd_155282, icsd_172468);
2. missing attribute values (one of the values of "_atom_site_attached_hydrogens" is missed in icsd_416608, one of the values of “_citation_page_*” is missed in icsd_39668, icsd_88900, icsd_63353 and icsd_173624, and one of the values of "_atom_site_B_iso_or_equiv" is missed in icsd_108810, icsd_151827, icsd_161952, icsd_161954, icsd_173447, and icsd_173448);
3. excessive blank lines or line break (appeared in icsd_413254, icsd_174306);
4. characters are not supported by UTF-8 (appeared in icsd_616316, icsd_042064, icsd_653637 and icsd_240772).

After manually correcting all of the repairable format errors, we saved the data to our platform and performed a procedure to prune the data:

1. Based on the values of “_atom_site_label” (VASL) and “_atom_type_symbol” (VATS), CIFs that VASL is not equal to VATS are removed, and CIFs where Li, Na, Mg and Al are neither in VASL nor VATS are removed, too. After that, 26,743 CIFs remain.
2. CIFs that contain only one element in VATS are removed. After that, 26,681 CIFs remain.
3. CIFs that contain hydrogen or its isotope in VATS are removed. After that, 23,023 CIFs remain.
4. CIFs that only contain zero-valence ions in VATS are removed. After that, 17,239 non-alloy CIFs remain.
5. CIFs that value of “_atom_site_occupancy" (VASE) is unequal to 1.0 (set tolerance as 0.0001). After that, 6,988 fully occupied compounds remain.

After that, error files captured by CAVD (during the ion-transport calculation) are also be deleted:

1. Some CIFs containing symmetrical equivalent sites (such as, the fractional coordinates of Li2 and N1 in icsd_066739 are symmetrical equivalent) are removed. After that, 6958 CIFs remain.
2. Some CIFs containing undetermined element positions (such as, the fractional coordinates of C in icsd_36483 are undetermined) are removed. After that, 6955 CIFs remain.

Eventually, 6,955 CIFs having Li (1,920 items), Na (2,841 items), Mg (1,125 items), and Al (1,743 items) are left for tool validation and ionic transport descriptors calculation.

## Recovery rate of the 2% exception compounds

We use the distance (*dis*) between mobile ion site and the nearest interstice to evaluate the recovery state of the mobile ion site: if *dis* <= *t*, the corresponding mobile ion is considered to be successfully recovered. The ratio of the number of mobile ions successfully recovered to the total number of mobile ions is then calculated as the recovery rate of the compound. Based on *t* = 0.5Å, >98% of the 6,955 compounds were complete recovery, and about 2% (Table S1) cannot be completely recovered (include ~1% partial recovery and ~1% miss compounds) by our model. The value of *dis* in some compounds (such as Li1 of icsd_001044, Li3 and Li6 of icsd_083986, and Na1 and Na4 of icsd_174310) are just slightly larger than the threshold 0.5Å. If the threshold is increased, those compounds are also completely recovered. When we increase the threshold to 0.6Å, the rate of complete recovery for Li^+^ is increased to 99.32%, Na^+^ to 99.40%, Mg^2+^ to 99.47%, and Al^3+^ to 99.77%. As for the remaining compounds with larger *dis* value (*dis* > 0.7Å), we found that some of them have two types of cations which may act as mobile ions, such as icsd_092312, icsd_092314, and icsd_092316 containing both Li^+^ and Na^+^. But in this recovery rate calculation, we only consider one of them as mobile ion, and the other as framework ion, which may affect recovery rate.

**Table S1.** Details of the recovery state about the 2% exceptions.

| Filename | *dis* | Recovery rate | |
| --- | --- | --- | --- |
|  |  | *t*=0.5Å | *t*=0.6Å |
| Li/icsd_001044.cif | {'Li1': 0.51} | 0 | 1 |
| Li/icsd_042327.cif | {'Li1': 0.56} | 0 | 1 |
| Li/icsd_059305.cif | {'Li1': 0.52} | 0 | 1 |
| Li/icsd_064962.cif | {'Li1': 0.59} | 0 | 1 |
| Li/icsd_069300.cif | {'Li1': 0.63} | 0 | 0 |
| Li/icsd_083832.cif | {'Li1': 0.51} | 0 | 1 |
| Li/icsd_092316.cif | {'Li1': 0.90} | 0 | 0 |
| Li/icsd_093015.cif | {'Li1': 0.51} | 0 | 1 |
| Li/icsd_107932.cif | {'Li1': 0.55} | 0 | 1 |
| Li/icsd_163219.cif | {'Li1': 0.64} | 0 | 0 |
| Li/icsd_245964.cif | {'Li1': 0.55} | 0 | 1 |
| Li/icsd_247089.cif | {'Li1': 0.56} | 0 | 1 |
| Li/icsd_415153.cif | {'Li1': 0.55} | 0 | 1 |
| Li/icsd_659706.cif | {'Li1': 1.19} | 0 | 0 |
| Li/icsd_002106.cif | {'Li1': 0.79, 'Li2': 0.43, 'Li3': 0.97} | 0.33 | 0.33 |
| Li/icsd_001045.cif | {'Li1': 0.32, 'Li2': 0.54} | 0.5 | 1 |
| Li/icsd_004201.cif | {'Li1': 0.09, 'Li2': 0.54} | 0.5 | 1 |
| Li/icsd_023477.cif | {'Li1': 0.62, 'Li2': 0.21} | 0.5 | 0.5 |
| Li/icsd_043689.cif | {'Li1': 0.00, 'Li2': 0.57} | 0.5 | 1 |
| Li/icsd_051442.cif | {'Li1': 0.04, 'Li2': 0.58} | 0.5 | 1 |
| Li/icsd_091284.cif | {'Li1': 0.63, 'Li2': 0.37} | 0.5 | 0.5 |
| Li/icsd_092468.cif | {'Li1': 0.22, 'Li2': 0.59} | 0.5 | 1 |
| Li/icsd_098845.cif | {'Li1': 0.51, 'Li2': 0.48} | 0.5 | 1 |
| Li/icsd_174101.cif | {'Li1': 0.10, 'Li2': 0.70} | 0.5 | 0.5 |
| Li/icsd_092314.cif | {'Li1': 0.58, 'Li2': 0.08, 'Li3': 0.09, 'Li4': 0.76, 'Li5': 0.23} | 0.6 | 0.8 |
| Li/icsd_083986.cif | {'Li1': 0.35, 'Li2': 0.38, 'Li3': 0.52, 'Li4': 0.46, 'Li5': 0.39, 'Li6': 0.55} | 0.67 | 1 |
| Li/icsd_092312.cif | {'Li1': 1.09, 'Li2': 0.03, 'Li3': 0.20} | 0.67 | 0.67 |
| Li/icsd_167238.cif | {'Li1': 0.22, 'Li2': 0.33, 'Li3': 0.69} | 0.67 | 0.67 |
| Li/icsd_247255.cif | {'Li1': 0.25, 'Li2': 0.56, 'Li3': 0.42, 'Li4': 0.03} | 0.75 | 1 |
| Li/icsd_247256.cif | {'Li1': 0.23, 'Li2': 0.56, 'Li3': 0.41, 'Li4': 0.03} | 0.75 | 1 |
| Li/icsd_051630.cif | {'Li1': 0.07, 'Li2': 0.04, 'Li3': 0.56, 'Li4': 0.05, 'Li5': 0.04, 'Li6': 0.02} | 0.83 | 1 |
| Li/icsd_096123.cif | {'Li1': 0.16, 'Li2': 0.32, 'Li3': 0.25, 'Li4': 0.32, 'Li5': 0.41, 'Li6': 0.54} | 0.83 | 1 |
| Li/icsd_157654.cif | {'Li1': 0.14, 'Li2': 0.19, 'Li3': 0.27, 'Li4': 0.38, 'Li5': 0.62, 'Li6': 0.23, 'Li7': 0.13} | 0.86 | 0.86 |
| Li/icsd_420126.cif | {'Li1': 0.30, 'Li2': 0.13, 'Li3': 0.06, 'Li4': 0.09, 'Li5': 0.26, 'Li6': 0.13, 'Li7': 0.21, 'Li8': 0.60} | 0.88 | 1 |
| Li/icsd_074950.cif | {'Li1': 0.09, 'Li2': 0.09, 'Li3': 0.08, 'Li4': 0.08, 'Li5': 0.09, 'Li6': 0.09, 'Li7': 0.12, 'Li8': 1.09, 'Li9': 0.14} | 0.89 | 0.89 |
| Na/icsd_024235.cif | {'Na1': 0.23, 'Na2': 0.60} | 0.5 | 1 |
| Na/icsd_033513.cif | {'Na1': 0.04, 'Na2': 0.63} | 0.5 | 0.5 |
| Na/icsd_033514.cif | {'Na1': 0.03, 'Na2': 0.62} | 0.5 | 0.5 |
| Na/icsd_062066.cif | {'Na1': 0.82, 'Na2': 0.17} | 0.5 | 0.5 |
| Na/icsd_063149.cif | {'Na1': 0.58, 'Na2': 0.00} | 0.5 | 1 |
| Na/icsd_066405.cif | {'Na1': 0.00, 'Na2': 0.68} | 0.5 | 0.5 |
| Na/icsd_073211.cif | {'Na1': 0.60, 'Na2': 0.09} | 0.5 | 0.5 |
| Na/icsd_073278.cif | {'Na1': 0.68, 'Na2': 0.00} | 0.5 | 0.5 |
| Na/icsd_402081.cif | {'Na1': 0.53, 'Na2': 0.00} | 0.5 | 1 |
| Na/icsd_412972.cif | {'Na1': 0.55, 'Na2': 0.00} | 0.5 | 1 |
| Na/icsd_412977.cif | {'Na1': 0.52, 'Na2': 0.00} | 0.5 | 1 |
| Na/icsd_412978.cif | {'Na1': 0.55, 'Na2': 0.00} | 0.5 | 1 |
| Na/icsd_008161.cif | {'Na1': 0.83, 'Na2': 0.42, 'Na3': 0.29} | 0.67 | 0.67 |
| Na/icsd_016654.cif | {'Na1': 0.09, 'Na2': 0.04, 'Na3': 1.19} | 0.67 | 0.67 |
| Na/icsd_021058.cif | {'Na1': 0.08, 'Na2': 0.45, 'Na3': 0.82} | 0.67 | 0.67 |
| Na/icsd_024419.cif | {'Na1': 0.09, 'Na2': 0.28, 'Na3': 0.65} | 0.67 | 0.67 |
| Na/icsd_033943.cif | {'Na1': 0.01, 'Na2': 0.33, 'Na3': 0.52} | 0.67 | 1 |
| Na/icsd_050665.cif | {'Na1': 0.14, 'Na2': 0.38, 'Na3':0.58} | 0.67 | 1 |
| Na/icsd_062316.cif | {'Na1': 0.46, 'Na2': 0.86, 'Na3':0.48} | 0.67 | 0.67 |
| Na/icsd_074173.cif | {'Na1': 0.06, 'Na2': 0.24, 'Na3': 0.53} | 0.67 | 1 |
| Na/icsd_001410.cif | {'Na1': 0.35, 'Na2': 0.62, 'Na3': 0.34, 'Na4': 0.38} | 0.75 | 0.75 |
| Na/icsd_174310.cif | {'Na1': 0.56, 'Na2': 0.06, 'Na3': 0.03, 'Na4': 0.55, 'Na5': 0.20, 'Na6': 0.16, 'Na7': 0.48, 'Na8': 0.27} | 0.75 | 1 |
| Na/icsd_411243.cif | {'Na1': 0.22, 'Na2': 0.22, 'Na3': 0.65, 'Na4': 0.27} | 0.75 | 0.75 |
| Na/icsd_416170.cif | {'Na1': 0.19, 'Na2': 0.75, 'Na3': 0.10, 'Na4': 0.17} | 0.75 | 0.75 |
| Na/icsd_092316.cif | {'Na1': 0.23, 'Na2': 0.22, 'Na3': 1.30, 'Na4': 0.14, 'Na5': 0.14} | 0.8 | 0.8 |
| Na/icsd_096596.cif | {'Na1': 0.18, 'Na2': 0.22, 'Na3': 0.25, 'Na4': 0.11, 'Na5':0.27, 'Na6': 0.38, 'Na7': 0.22, 'Na8': 0.93, 'Na9': 0.40, 'Na10': 0.21, 'Na11': 0.53, 'Na12': 0.45} | 0.83 | 0.92 |
| Na/icsd_032600.cif | {'Na1': 0.56, 'Na2': 0.10, 'Na3': 0.11, 'Na4': 0.16, 'Na5': 0.14, 'Na6': 0.10, 'Na7': 0.27, 'Na8': 0.30, 'Na9': 0.13} | 0.89 | 1 |
| Na/icsd_039237.cif | {'Na1': 0.15, 'Na2': 0.10, 'Na3': 0.03, 'Na4': 0.11, 'Na5': 0.02, 'Na6': 0.03, 'Na7': 1.32, 'Na8': 0.01, 'Na9': 0.07} | 0.89 | 0.89 |
| Na/icsd_412212.cif | {'Na1': 0.51, 'Na2': 0.30, 'Na3': 0.08, 'Na4': 0.10, 'Na5': 0.21, 'Na6': 0.30, 'Na7': 0.11, 'Na8': 0.38, 'Na9': 0.23} | 0.89 | 1 |
| Na/icsd_098709.cif | {'Na1': 0.10, 'Na2': 0.25, 'Na3': 0.13, 'Na4': 0.37, 'Na5': 0.21, 'Na6': 0.16, 'Na7': 0.28, 'Na8': 0.07, 'Na9': 0.15, 'Na10': 0.04, 'Na11': 0.36, 'Na12': 0.13, 'Na13': 0.19, 'Na14': 0.53, 'Na15': 0.30, 'Na16': 0.18, 'Na17': 0.40, 'Na18': 0.22, 'Na19': 0.09, 'Na20': 0.43, 'Na21': 0.16, 'Na22': 0.20, 'Na23': 0.25, 'Na24': 0.26} | 0.96 | 1 |
| Mg/icsd_051246.cif | {'Mg1': 0.96} | 0 | 0 |
| Mg/icsd_066952.cif | {'Mg1': 0.90} | 0 | 0 |
| Mg/icsd_066953.cif | {'Mg1': 0.74} | 0 | 0 |
| Mg/icsd_087116.cif | {'Mg1': 0.85} | 0 | 0 |
| Mg/icsd_087117.cif | {'Mg1': 0.86} | 0 | 0 |
| Mg/icsd_280938.cif | {'Mg1': 0.52} | 0 | 1 |
| Mg/icsd_404851.cif | {'Mg1': 0.53} | 0 | 1 |
| Mg/icsd_033689.cif | {'Mg1': 0.0, 'Mg2': 0.61} | 0.5 | 0.5 |
| Al/icsd_056821.cif | {'Al1': 1.15} | 0 | 0 |
| Al/icsd_081572.cif | {'Al1': 0.52} | 0 | 1 |
| Al/icsd_027596.cif | {'Al1': 0.12, 'Al2': 0.63} | 0.5 | 0.5 |
| Al/icsd_036324.cif | {'Al1': 0.12, 'Al2': 0.63} | 0.5 | 0.5 |
| Al/icsd_245730.cif | {'Al1': 0.38, 'Al2': 0.78} | 0.5 | 0.5 |
| Al/icsd_016970.cif | {'Al1': 0.024, 'Al2': 0.54, 'Al3': 0.20, 'Al4': 0.21} | 0.75 | 1 |

## Descriptions of the data in Descriptors.xlsx, VestaFiles.zip and Channels.zip

***Symmetry_space_group_name_H-M***: The name of symmetry space group, which is extracted from the CIF.

***R_T_***: The size of restricting interstice or bottleneck in the interstitial network, *R_T_*, characterizes the radius of largest ion that can freely pass through the void space.

***R_Ta_***, ***R_Tb_***, ***R_Tc_***: The RT for three crystallographic directions: *R_Ta_* (*a*), *R_Tb_* (*b*), and *R_Tc_* (*c*).

***IND***: The dimension of the interstitial network.

***TCD***: A list containing the dimension of the connected transport channel. If no connected channels are acquired, the *TCD* is empty. Due to the transport network may consist of more than one channel, the dimension of a single channel may differ from the dimension of the transport network.

***Acc***: The value of *Acc_a_*, *Acc_b_*, and *Acc_c_* can be determined by *R_Ta_*, *R_Tb_*, and *R_Tc_* with *T_l_*, respectively. For example, if *R_Ta_* >= *T_l_*, *Acc_a_* = Ture; otherwise, *Acc_a_* = False.

***Recovery rate***: The mobile ion recovery rate for each CIF.

***Ionic_radii***: The calculated ionic radii for radical Voronoi decomposition. This value is obtained by combining the rigorous definition of coordination number proposed by O'Keeffe^2,3^ and the table of the effective ionic radii of Shannon^4^.

***Minimal_mobile-framework_distance***: The list of minimal mobile-framework distances calculated in different coordination environments. Each item of the list is a tuple, which the first item is a coordination number, and the second item is a distance tuple. The first item of the distance tuple is the distance from the center of mobile ion to the center of nearest framework ion, and the second item is the distance between the center of mobile ion and the surface of nearest framework ion.

***.vesta file**: This file is a structure file for visualizing the transport channel (by VESTA^5^). The interstices and bottlenecks of the channels are represented by the atoms “He” and “Ne”, respectively, and the channel segment between the two interstices is represented by the bond between atoms “He”. In addition, the radii of atoms “He” and “Ne” are set as the radii of the interstice and bottleneck, and the length of the bond is set as the length of the channel segment. In *.vesta file, the coordinates of the interstice and bottleneck are provided in symmetry group *P*1. *.vesta file will not be provided for compounds without any channels.

***.net file**: This file is a record of the calculated transport channels. Each channel is recorded by five parts: channel id, the dimensionality of the channel, the lattice of the unit cell, the interstitial table, and the connection table. The information listed in the interstitial table are id, symmetrical label (symmetrically equivalent interstices have same label), fractional coordinates, radius. The information listed in the connection table are id of the source interstice, id of the sink interstice, periodic displacement vector of the sink interstice, fractional coordinates of the related bottleneck, the radius of the related bottleneck, and the length of the channel segment. *net file will not be provided for compounds without any channels.

## Results of four typical structures

LiFePO_4_ is an olivine-structured orthophosphate which has been used as a cathode material due to its environment-friendliness and low cost of Fe element.^6^ After the calculation of transport channel, a 1D transport channel consisting of 8 symmetrically distinct interstices and 7 different channel segments (Table S2, Figure S4 (a)) is obtained. In the channel, paths It3-It6-It3 form the main part of the transport channel, where It3 coincides with experimentally determined Li1 within 0.1227Å. Therefore, the migration path of Li-ion can be regarded as Li1-It6-Li1, which is along the direction of b-axis and coincides with the results of BVSE calculation and *ab initio*.^6,7^

**Table S2.** The sites of symmetrically distinct interstices and the channel segments between them in LiFePO_4_ (icsd_56291).

| Interstitial label | Interstitial site | Channel segments |
| --- | --- | --- |
| It1 | (0.3033, 0.2500, 0.5243) | It1-It2, It1-It4, It1-It6 |
| It2 | (0.1533, 0.5342, 0.8928) | It2-It1 |
| It3 | (0.0058, 0.5120, 0.0201) | It3-It5, It3-It6 |
| It4 | (0.2138, 0.7500, 0.0512) | It4-It1, It4-It8 |
| It5 | (0.0114, 0.4944, 0.0003) | It5-It3 |
| It6 | (0.0944, 0.7500, 0.1251) | It6-It1, It6-It3, It6-It7 |
| It7 | (0.0915, 0.7500, 0.2549) | It7-It6 |
| It8 | (0.2155, 0.7500, 0.0965) | It8-It4 |

Na β-Al_2_O_3_ (Na_2_O(Al_2_O_3_)_11_) is a widely used as solid electrolyte in sodium–sulfur battery and acts as a separator material between the two electrodes.^8^ In the structure of Na β-Al_2_O_3_, O^2-^ forms a cubic close packing, and Al^3+^ occupies the octahedron and tetrahedral interstices. The four layers of closely packed Al-O polyhedron form a base block with a loosely packed Na-O layer between the blocks. Therefore, Na^+^ is liable to migrate in the Na-O layer perpendicular to the *c*-axis direction. Our result, a 2D transport channel perpendicular to the *c*-axis with 4 symmetrically distinct interstices and 4 channel segments between the interstices (Table S3, Figure S4 (b)), also confirms the previous conclusions.^9^

**Table S3.** The sites of symmetrically distinct interstices and the channel segments between them in Na_2_O(Al_2_O_3_)_11_ (icsd_60635).

| Interstitial label | Interstitial site | Channel segments |
| --- | --- | --- |
| It1 | (0.3010, 0.1504, 0.2500) | It1- It1', It1-It2 |
| It2 | (0.8333, 0.16667, 0.2453) | It2-It1, It2-It3 |
| It3 | (0.3982, 0.1991, 0.2500) | It3-It2, It3-It3, It3-It4 |
| It4 | (0.6667, 0.3333, 0.2500) | It4-It3 |

| 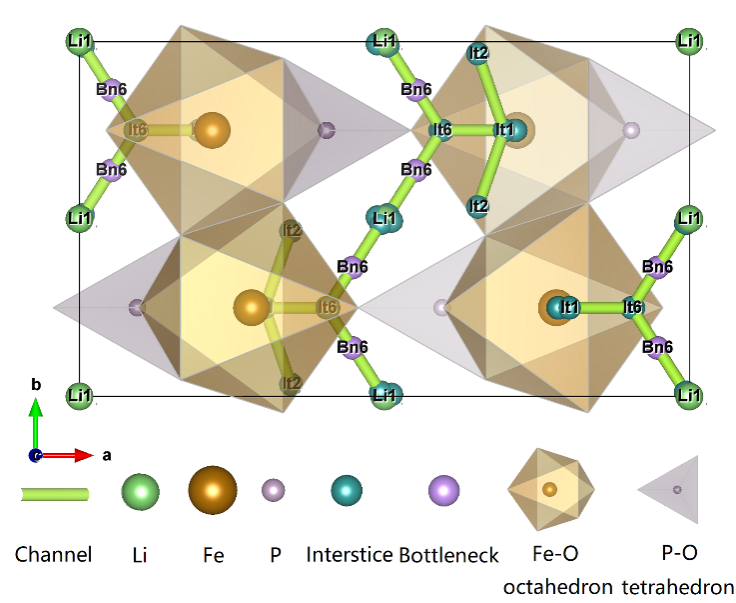 | 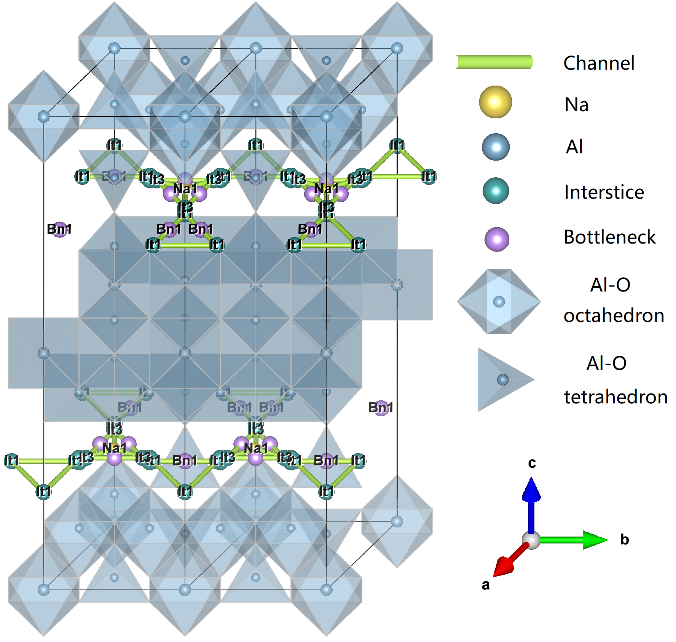 |
| --- | --- |
| (a) | (b) |

**Figure S4.** The calculated transport channels of Li^+^ in LiFePO_4_ **(a)** and Na^+^ in Na β-Al_2_O_3_ (**b**). **(a)** The 1D Li^+^ channel in LiFePO_4_. All of interstices included in the channel are connected each other (see VestaFiles/icsd_56291.vesta in figshare), but part of them are omitted in the figure for clarity. **(b)** The 2D Na^+^ layer channel in Na β-Al_2_O_3_. It4 is consistent with Na1; It3 is connected to its symmetrical equivalent site in same unit cell; It1 is connected to its periodic image in adjacent unit cell (see VestaFiles/icsd_60635.vesta in figshare).

**Table S4.** The sites of symmetrically distinct interstices and the channel segments between them in Na_4_Zr_2_(SiO_4_)_3_ (icsd_38055).

| Interstitial label | Interstitial site | Channel segments |
| --- | --- | --- |
| It1 | (0.9573, 0.5992, 0.5586) | It1-It2, It1-It3, It1-It10 |
| It2 | (0.9357, 0.5794, 0.5741) | It2-It1, It2-It5, It2-It10 |
| It3 | (0.9741, 0.6402, 0.5981) | It3-It1, It3-It4, It3-It5, It3-It8, It3-It10, It3-It11, It3-It12 |
| It4 | (0.9526, 0.7409, 0.5109) | It4-It3, It4-It7, It4-It12 |
| It5 | (0.9499, 0.6165, 0.5833) | It5-It2, It5-It3, It5-It10, It5-It11 |
| It6 | (1.0, 0.0, 0.0) | It6-It7 |
| It7 | (0.2042, 0.1834, 0.9826) | It7-It4, It7-It6, It7-It8, It7-It12 |
| It8 | (0.3331, 0.2993 0.9226) | It8-It3, It8-It7, It8-It9, It8-It11, It8-It12, It8-It13 |
| It9 | (0.03396, 0.7006, 0.5833) | It9-It8, It9-It11, It9-It13 |
| It10 | (0.9423, 0.6208 0.5955) | It10-It1, It10-It2, It10-It3, It10-It5 |
| It11 | (0.9999, 0.6665, 0.5833) | It11-It3, It11-It5, It11-It8, It11-It9 |
| It12 | (0.2829, 0.2800, 0.9496) | It12-It3, It12-It4, It12-It7, It12-It8 |
| It13 | (0.3331, 0.2992, 0.9206) | It13-It8, It13-It9 |

**Table S5.** The sites of symmetrically distinct interstices and the channel segments between them in Li_7_La_3_Zr_2_O_12_ (icsd_246817).

| Interstitial label | Interstitial site | Channel segments |
| --- | --- | --- |
| It1 | (0.5751, 0.1481, 0.9597) | It1-It2, It1-It3, It1-It21 |
| It2 | (0.5833, 0.1366, 0.9699) | It2-It1, It2-It9, It2-It21 |
| It3 | (0.9202, 0.2080, 0.9969) | It3-It1, It3-It15, It3-It21 |
| It4 | (0.3318, 0.2422, 0.0493) | It4-It7, It4-It14, It4-It22 |
| It5 | (0.2913, 0.0024, 0.0830) | It5-It6, It5-It17, It5-It24 |
| It6 | (0.3514, 0.0438, 0.0790) | It6-It5, It6-It16, It6-It24 |
| It7 | (0.1729, 0.2101, 0.8968) | It7-It4, It7-It18, It7-It22 |
| It8 | (0.9742, 0.0589, 0.8976) | It8-It18 |
| It9 | (0.1062, 0.3129, 0.9551) | It9-It2, It9-It13, It9-It15, It9-It18, It9-It20, It9-It21 |
| It10 | (0.1997, 0.4497, 0.1250) | It10-It12, It10-It17, It10-It23 |
| It11 | (0.2500, 0.3812, 0.0000) | It11-It12, It11-It13 |
| It12 | (0.1953, 0.4501, 0.1099) | It12-It10, It12-It11, It12-It17, It12-It23 |
| It13 | (0.3484, 0.3131, 0.0592) | It13-It9, It13-It11, It13-It14, It13-It16, It13-It20 |
| It14 | (0.1486, 0.2730, 0.9389) | It14-It4, It14-It13, It14-It15, It14-It18, It14-It20, It14-It22 |
| It15 | (0.1038, 0.3046, 0.9552) | It15-It3, It15-It9, It15-It14, It15-It19, It15-It20, It15-It21 |
| It16 | (0.2843, 0.3883, 0.1625) | It16-It6, It16-It13, It16-It17, It16-It24 |
| It17 | (0.2094, 0.4448, 0.1391) | It17-It5, It17-It10, It17-It12, It17-It16,It17-It23, It17-It24 |
| It18 | (0.3310, 0.2148, 0.1090) | It18-It7, It18-It8, It18-It9, It18-It14, It18-It22 |
| It19 | (0.0000, 0.2500, 0.3750) | It19-It15 |
| It20 | (0.3724, 0.6991, 0.4474) | It20-It9, It20-It13, It20-It14, It20-It15 |
| It21 | (0.4103, 0.6750, 0.4955) | It21-It1, It21-It2, It21-It3, It21-It9, It21-It15 |
| It22 | (0.1647, 0.7650, 0.5807) | It22-It4, It22-It7, It22-It14, It22-It18 |
| It23 | (0.2969, 0.5534, 0.6347) | It23-It10, It23-It12, It23-It17 |
| It24 | (0.3274, 0.9900, 0.4099) | It24-It5, It24-It6, It24-It16, It24-It17 |

## References

1. The Inorganic Crystal Structure Database https://icsd.fiz-karlsruhe.de/search/ (2010).

2. O’Keeffe, M. A proposed rigorous definition of coordination number. *Acta Cryst. A* **35**, 772–775 (1979).

3. Ong, S. P. *et al.* Python Materials Genomics (pymatgen): A robust, open-source python library for materials analysis. *Comput. Mater. Sci.* **68**, 314–319 (2013).

4. R.D.Shannon. Revised Effective Ionic Radii and Systematic Studies of Interatomie Distances in Halides and Chaleogenides. *Acta Cryst. A* **32**, 751–767 (1976).

5. Momma, K. & Izumi, F. VESTA: A three-dimensional visualization system for electronic and structural analysis. *J. Appl. Crystallogr.* **41,** 653–658 (2008).

6. Islam, M. S. & Fisher, C. A. J. Lithium and sodium battery cathode materials: computational insights into voltage, diffusion and nanostructural properties. *Chem*. *Soc*. *Rev*. **43,** 185–204 (2014).

7. Chen, D. *et al.* High throughput identification of Li ion diffusion pathways in typical solid state electrolytes and electrode materials by BV-Ewald method. *J. Mater. Chem. A* **7**, 1300–1306 (2019).

8. Song, S., Duong, H. M., Korsunsky, A. M., Hu, N. & Lu, L. A Na^+^ Superionic Conductor for Room-Temperature Sodium Batteries. *Sci*. *Rep*. **6,** 32330 (2016).

9. Yao, Y. -F. Y. & Kummer, J. T. Ion exchange properties of and rates of ionic diffusion in beta-alumina. *J. Inorg. Nucl. Chem.* **29**, 2453–2475 (1967).
